# Integrin αvβ3 as a Non-Genomic Estrogen Receptor in Breast Cancer for Signaling Pathways and Crosstalk

**DOI:** 10.3390/cells14221832

**Published:** 2025-11-20

**Authors:** Kuan Wang, Zi-Lin Li, Lin-Yi Huang, Chih-Jung Yao, Dana R. Crawford, Chih-Yang Wang, Ju-Ku Mo, Ya-Jung Shih, Hung-Yun Lin, Jacqueline Whang-Peng

**Affiliations:** 1 Graduate Institute of Nanomedicine and Medical Engineering, College of Medical Engineering, Taipei Medical University, Taipei 11031, Taiwan; wangk007@gmail.com (K.W.); lizilin919@tmu.edu.tw (Z.-L.L.); tracy451210@gmail.com (J.-K.M.); shihyj@tmu.edu.tw (Y.-J.S.); 2 Graduate Institute of Cancer Biology and Drug Discovery, College of Medical Science and Technology, Taipei Medical University, Taipei 11031, Taiwan; chihyang@tmu.edu.tw (C.-Y.W.); jqwpeng@nhri.edu.tw (J.W.-P.); 3 Department of Pediatrics, E-DA Hospital, I-Shou University, Kaohsiung 82445, Taiwan; pedptch05680@gmail.com; 4 Department of Internal Medicine, School of Medicine, College of Medicine, Taipei Medical University, Taipei 11031, Taiwan; yao0928@tmu.edu.tw; 5 Department of Medical Education and Research, Wan Fang Hospital, Taipei Medical University, Taipei 11696, Taiwan; 6 Department of Immunology and Microbial Disease, Albany Medical College, 43 New Scotland Avenue, Albany, NY 12208, USA; crawfod@amc.edu; 7 Ph.D. Program for Cancer Molecular Biology and Drug Discovery, College of Medical Science and Technology, Taipei Medical University, Taipei 11031, Taiwan; 8 TMU Research Center of Cancer Translational Medicine, Taipei Medical University, Taipei 11031, Taiwan; 9 Lung Cancer Research Team, Taipei Cancer Center, Taipei Medical University, Taipei 11031, Taiwan; 10 Traditional Herbal Medicine Research Center of Taipei Medical University Hospital, Taipei Medical University, Taipei 11031, Taiwan; 11 Cancer Center, Wan Fang Hospital, Taipei Medical University, Taipei 11031, Taiwan

**Keywords:** breast cancer, estrogen, estrogen receptor, GPER, integrin αvβ3

## Abstract

**Highlights:**

**What are the main findings?**

- Integrin αvβ3 functions as a crucial non-genomic receptor for estrogen, initiating rapid activation of FAK, ERK1/2, and PI3K signaling pathways.
- This non-genomic estrogen signaling is shown to critically modulate integrin αvβ3 activity, subsequently driving cancer proliferation, migration, and metastasis

**What is the implication of the main finding?**

- Crosstalk among estrogen, integrin αvβ3, and GPER generates diverse cellular effects relevant to breast cancer biology.
- Targeting the Integrin αvβ3 non-genomic axis is proposed as a therapeutic strategy to overcome resistance observed in cancers.

**Abstract:**

Integrin αvβ3, a key member of the integrin family, plays a crucial role in cell localization, mobilization, and signal transduction through collaborating with extracellular proteins. Its unique expression and activation in tumor cells and rapidly dividing endothelial cells suggest its potential role in cancer cell growth and metastasis, making it a promising therapeutic target. In genomic pathways, estrogen binds to its receptors to form transcription complexes that bind to the promoters of steroid hormone-receptive genes. Conversely, G protein-coupled estrogen receptor 1 (GPER) and integrin αvβ3 have been shown to play oles in non-genomic actions that contribute to estrogen-induced cancer growth. The molecular mechanisms of these non-genomic functions involve signal transduction via focal activated kinase (FAK), mitogen-activated protein kinase (ERK1/2), and phosphatidylinositol 3-kinase (PI3K), as well as the differential expression of multiple genes associated with various cellular processes. As a hormone receptor, integrin αvβ3, collaborating with ER-α and GPER, exhibits a wide range of cellular effects relevant to cancer biology.

## 1. Introduction

**Figure 1:**
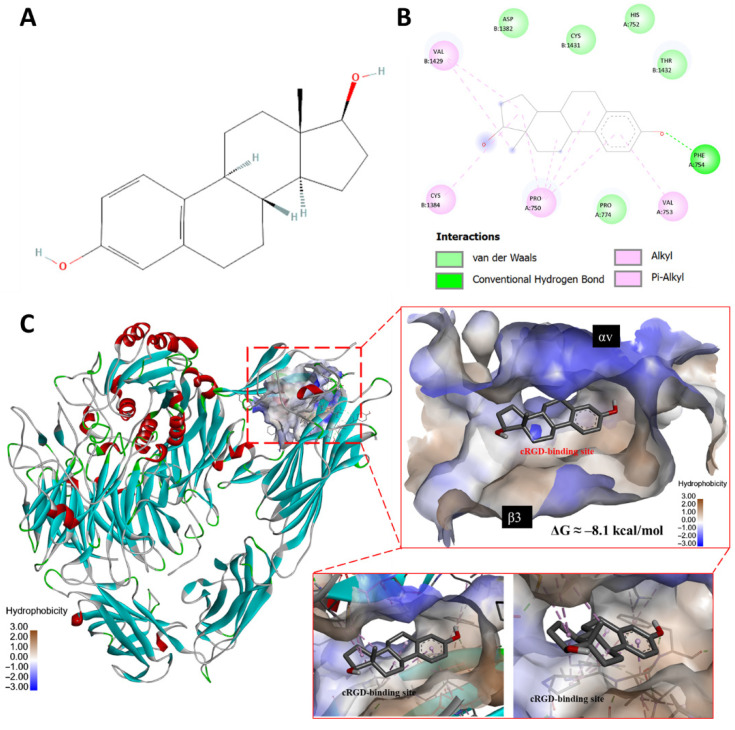
Molecular docking of estradiol with the cRGD-binding site of integrin αvβ3. (**A**) Two-dimensional structure of estradiol (PubChem CID: 5757). (**B**) Two-dimensional interaction diagram of estradiol docked into integrin αvβ3, showing key contacts with surrounding residues. Interaction types are indicated as van der Waals (light green), conventional hydrogen bonds (bright green), and alkyl/π-alkyl interactions (pink). Major interacting residues include Asp1382, His752, Thr1432, Val1429, Cys813, Pro750, and Phe754. (**C**) Three-dimensional docking analysis of estradiol in integrin αvβ3 (PDB ID: 1L5G). The protein is shown in docking representation, colored according to hydrophobicity (hydrophilic = blue; hydrophobic = brown). Estradiol (black stick model) is bound within the canonical cRGD-binding pocket located at the interface between the αv β-propeller domain (labeled αv, upper surface) and the β3 βA domain (labeled β3, lower surface). The β3 domain harbors the metal ion-dependent adhesion site (MIDAS), which typically coordinates the Asp of the RGD motif; in this docking, estradiol occupies an overlapping groove. The predicted binding free energy was ΔG ≈ −8.1 kcal/mol, consistent with stable binding. Insets provide close-up surface views, highlighting estradiol in the cRGD-binding site.

Breast cancer is among the most prevalent and clinically significant malignancies, characterized by diverse morphological and molecular characteristics. Estrogen receptor-α (ER-α) promotes signal transduction in breast cancer cells [1]. Through its nuclear translocation and activation of ER-α, estrogen plays a crucial role in regulating various biological functions, including sexual organ development, pregnancy, bone density, cholesterol recruitment, brain function, and the cardiovascular system [1,2,3]. The estrogen receptor complex translocates into the nucleus, disengaging corepressors and attracting coactivator proteins to modulate gene expression. In addition, breast cancer cells express ER-β; ER-α and ER-β have distinct biological functions and often counteract each other’s effects [4]. ER-α and ER-β appear to have overlapping but unique downstream target genes. Thus, ER-α and ER-β have different transcriptional activities in specific ligand, cell-type, and promoter contexts [5]. ER-β has been shown to regulate cancer cell proliferation and suppress breast cancer growth [6]. Studies have demonstrated that a cell surface receptor, integrin αvβ3, acts as a receptor for androgen [7,8,9] and progesterone [10] in triple-negative breast cancer cells. Estrogen contains a steroid bone structure and molecule docking modeling shown in publications, and Figure 1 indicates that estrogen fits into the RGD pocket in the integrin αvβ3. Additionally, the integrin αvβ3 antagonist, RGD, influences cell motility and adhesion in primary human breast cancer cultures [11], and estrogen affects integrin αvβ3-dependent cellular activities [12]. These evidences suggest that integrin αvβ3 is a binding site of estrogen and plays a role in estrogen-dependent activity.

On the other hand, nongenomic effects of steroid hormones begin at the hormone receptor on the cell surface, triggering signal transduction pathways. In addition, G protein-coupled estrogen receptor 1 (GPER) and integrins play essential roles in signal transduction and cellular bioactivities. Those signal pathways include FAK, ERK1/2, and PI3K, which crosstalk with each other [13,14]. In this review, we discuss the functions of integrin αvβ3, the interactions among estrogen, GPER, and integrin αvβ3, and the downstream biological consequences of the interactions. Additionally, potential therapeutic candidates for blocking these interactions will be briefly discussed.

## 2. Integrin αv- and β3-Related Integrins in Breast Cancers

Several αv- and β3-related integrins are expressed in breast cancer cells with different functions in breast cancer growth and metastasis (Table 1). Integrins β1 and αv are highly expressed in breast cancer cells, whereas breast cancer cells also express higher levels of integrin β5 and integrin αvβ5 [15]. Integrin αvβ3 is highly expressed in breast cancer cells compared to normal breast epithelial cells [16].

MDA-MB-435 cells consistently expressed higher levels of integrin β3 and integrin αvβ3 [15,17]. Various integrins contribute differentially to ER-positive and ER-negative breast cancer cells. Cohort analysis studies of differential gene expression in breast cancers reveal a strong correlation between high integrin β3 expression and early metastasis, as well as shorter disease-free survival, in patients with ER-negative tumors [18]. On the other hand, increased expression of integrin αv is frequently associated with tumor cell adhesion, migration, invasion, and metastasis. It also correlates with poor prognosis in breast cancer [19]. The transcriptional factor Forkhead box class O (FoxO)3a induces integrin α5 expression to inhibit tamoxifen-resistant breast cancer progression [17]. FoxO3a reduces the motility and invasiveness of tamoxifen-resistant (TamR) breast cancer cells via the induction of the integrin α5 subunit of the fibronectin receptor α5β1 [17]. A strong positive linkage between FoxO3a and integrin α5 has been demonstrated in ER-α-positive, but not in ER-α-negative, breast cancer patients [17]. Early integrin β3 expression in breast cancer is vital and required for the spontaneous dissemination of bone-metastatic breast cancer [18]. Integrin αvβ3 is the cell surface receptor of resveratrol [8] and doxycycline-induced antiproliferation in breast cancer cells [20]. Additionally, integrin αvβ3 mediates dihydrotestosterone-induced proliferation of breast cancer cells [21]. Integrin β1 is associated with the membrane and follows the same endocytosis and subcellular trafficking pathway triggered by estrogen [22]. Moreover, ER-α-positive cysts are present within human breast tissues, and their colocalization with β1 integrin is primarily detected in tumors [22]. Estrogen induces ER-α-positive breast cancer aggressiveness by promoting cell proliferation and survival, the epithelial–mesenchymal transition, and acquiring stem-like properties. Integrin β4 signaling has been implicated in estrogen/ER-α-induced tumorigenicity and anti-apoptosis. Estrogen enhances ER-α-positive breast cancer cell viability and motility through activating ΔNp63, an N-terminally truncated isoform of the p63 transcription factor, through the ERα-ΔNp63 integrin β4 signaling pathway, to induce AKT phosphorylation [21]. Tumor metastasis is the leading cause of cancer-associated mortality. Unfortunately, the underlying mechanism of metastasis remains poorly understood. Triple-negative breast cancer is characterized by aggressive biological features, which allow relapse and metastatic spread to occur more frequently than in hormone receptor-positive subtypes [23]. Extracellular connective tissue growth factor (CTGF) can directly bind to integrin αvβ3, stimulating the FAK/Src/nuclear factor κ-light-chain-enhancer of activated B cells (NF-κB) p65 signaling axis, which results in the upregulation of glucose transporter 3 (Glut3) transcription. Neutralization of CTGF decreases cell proliferation, migration, and invasion by downregulating Glut3-mediated glycolytic phenotypes [24]. Expression of legumain, an endo-lysosomal cysteine protease, is positively correlated with metastatic progression and poor prognosis in breast cancer. A zymogenic form of legumain is secreted by motile breast cancer cells [25]. Through binding to cell surface integrin αvβ3 via an RGD motif, the autocrine pro-legumain activates FAK-Src-Ras homolog family member A (RhoA) signaling in cancer cells. Pro-legumain promotes cancer cell migration and invasion independent of legumain protease activity [25]. As previously mentioned, cytoplasmic FAK is a critical component of the integrin-mediated transduction pathway. It contributes to various signaling cascades triggered by stimuli such as growth factors and cytokines [26]. Moreover, the activation of FAK induced by estrogen in breast tumors involves the ER and the GPER [27]. The signaling is triggered by the biochemical properties of the integrin–ligand interaction and by the physical forces transferred from the cytoskeleton and the extracellular matrix (ECM). Integrins play various roles in different types and stages of cancer. However, monotherapy with ECM- and integrin-targeting agents has demonstrated limited clinical efficacy. Therefore, multitargeting therapeutic strategies are a more rational choice. Combining personalized precision medicine is expected to boost the development of effective therapeutic strategies [28].

The combination of paclitaxel with integrin αv’s knockdown is a more effective therapeutic option than using a variety of paclitaxel with cilengitide, an inhibitor for integrin αvβ3 and αvβ5, for triple-negative breast cancer [29]. However, combining αv-specific small interfering RNA (siRNA) or cilengitide with paclitaxel fails to inhibit the growth of melanoma cell lines due to reduced paclitaxel sensitivity [29]. Alternatively, the expression of tumor integrin β3 is essential for the early and influential spontaneous metastasis of breast cancer to the bone and soft tissue. Integrin β3 promotes migration, protease expression, and transendothelial migration in vitro. While in vivo, integrin increases vascular dissemination. However, animal studies are conflicting and indicate heterogeneity in the relative contributions of β3-expressing tumor and stromal cell populations in different types of cancer [18]. Consistent downregulation of tumor integrin β3 significantly impairs spontaneous but not experimental bone and lung metastases without affecting the growth of the primary breast tumor. Unlike subcutaneous tumors, the vasculature, development, and spontaneous metastasis of orthotopic tumors were unchanged in integrin β3-deficient mice. Although many preclinical studies have suggested that integrin β3 receptors (αvβ3 and αIIbβ3) play a role in cancer progression, integrin β3 inhibitors have shown limited efficacy in patients with advanced solid tumors [18]. The unreasonable ineffectiveness of integrin β3 inhibitors in patients could be due to an incomplete understanding of the precise function of integrin β3 and, consequently, inappropriate clinical application [18]. Integrin αvβ3 is shown to act as a cell surface receptor for estrogen [30]. In contrast, resveratrol, a stilbene, exhibits an estrogenic effect while inhibiting breast cancer proliferation, which has been demonstrated to bind to the integrin αvβ3 RGD binding domain [31]. Resveratrol inhibits cell proliferation in ER-positive and ER-negative breast cancer cells by acting through integrin αvβ3. In MCF-7 cells, estrogen promotes cell proliferation and partially counteracts the antiproliferative effects of resveratrol [32]. Furthermore, estrogen activates FAK [27,33], leading to the reorganization of actin in prostate and breast cancer cells via the FAK, PI3K, and Rac1 pathways [20,34]. Activated FAK contributes to Src homology and collagen (Shc) phosphorylation and is likely to promote rat sarcoma virus (Ras) activity, ERK1/2 activation, and cell proliferation [35].

**Table 1:** Various integrin αv- and β3-related integrins present in breast cancers.

Integrin	Cancer Type/Source	Cellular Functions
Integrin αv	Invasive breast carcinomas/patient samples and clinical data.	Expressed and localized in tumor cells; stimulates breast cancer invasion [36].
Integrin β3	Bone-metastatic breast cancer/mouse mammary tumor line	Essential for early spontaneous dissemination [18].
	Breast cancer cells/patient samples.	Interacts with IL-32/p38-MAPK to promote EMT and invasion [37].
Integrin αvβ1	Metastatic breast cancer cells/human breast cancer MCF10CA1a (CA1a) cells and mouse breast cancer 4T1 and 4TO7 cells.	Enriched in extracellular vesicles of metastatic breast cancer cells [38]. A mechanism mediated by galectin-3
Integrin αvβ3	ER-α-negative MDA-MB-231/cell line.	Regulates cell proliferation [7].
	ER-α-negative MDA-MB-231/cell line.	Stimulates the proliferation of ER-negative breast cancer cells [8].
	ER-α-negative MDA-MB-231/cell line.	Integrin αvβ3 is essential in doxycycline-induced antiproliferation in breast cancer cells [20].
	ER-α-negative breast cancer/cell line.	Regulates tumor cell migration [36].
	ER-α-negative MDA-MB-435/cell line.	Vitronectin/mTOR; IL-8/PI3K/Akt/NF-κB promotes tumor metastasis [39].
	ER-α-positive MCF-7/cell line.	CCN1/CYR61 binds to the integrin αvβ3 receptor, stimulate tumor growth, chemoresistance, and angiogenesis [40].
	HER2-positive breast cancer/murine brain metastatic TBCP-1 cells and human non-metastatic BT474 and SKBR3 cells.	Integrin αvβ3 is a master regulator of resistance to tyrosine kinase inhibitors (TKIs)-induced ferroptosis [41].
Integrin αvβ5	Breast cancer-exo/cell line.	S100 promotes the formation of a pre-metastasis niche [42].
Integrin αvβ6	Triple-negative breast cancer/human and murine TNBCs.	SOX4 transcription factor is an essential resistance mechanism against T cell-mediated cytotoxicity in triple-negative breast cancer cells (TNBC) [43]. Promotes an osteolytic program in cancer cells by upregulating matrix metalloproteinase 2 (MMP2). Induces cell adhesion and migration

## 3. Exploring a Novel Interaction Between Estrogen and Integrin αvβ3

The activity of integrin αvβ3 is closely linked to cancer progression, and numerous antagonists have been designed to target its RGD-binding site, thereby inhibiting proliferation and invasion [44]. Legumain, an endo-lysosomal cysteine protease, interacts with integrin αvβ3 through the RGD motif and has been shown to suppress breast cancer cell migration and invasion [25]. Other RGD-based therapeutic agents have also been investigated in breast cancer models [45,46]. While integrin αvβ3 is not strictly essential for ER-α-positive breast cancer proliferation, its canonical cRGD-binding pocket may allow direct interactions with estrogen, providing an alternative regulatory mechanism.

**Molecular Docking Analysis of Estradiol Binding to the cRGD Site of Integrin αvβ3.**

Molecular docking was performed to predict the interaction of estradiol with the cRGD-binding site of integrin αvβ3, using a protocol previously reported in Science [47]. The crystal structure of integrin αvβ3 in complex with cyclic RGD peptide (PDB ID: 1L5G) was obtained from the RCSB Protein Data Bank [47]. The protein was prepared by removing water molecules and co-crystallized ligands using PyMOL (v2.5), followed by adding polar hydrogens and Kollman charges in AutoDockTools (v1.5.7) [48]. The 3D structure of estradiol (PubChem CID: 5757) was retrieved from PubChem and energy-minimized using the MM2 force field in Chem3D. The binding pocket corresponding to the canonical cRGD-recognition groove at the αv–β3 interface was defined as the docking site, encompassing the metal ion-dependent adhesion site (MIDAS) region of the β3 subunit. The grid box parameters were built for integrin αvβ3 (center: x = 16, y = 43, z = 47; size: x × y × z = 45 × 45 × 45). Docking simulations were conducted using AutoDock Vina (v1.2.3) with an exhaustiveness value of 8 and an energy range of 4 to ensure thorough sampling of conformational space. The binding free energies (ΔG, kcal/mol) were calculated for the resulting protein–ligand affinities.

The docking analysis demonstrates that estradiol (E_2_) can be accommodated within the cRGD-binding pocket of integrin αvβ3, as illustrated in Figure 1. This pocket is located at the interface between the αv subunit (β-propeller domain, represented in blue) and the β3 subunit (βA domain, represented in brown). A defining feature of this region is the MIDAS motif of β3, which coordinates divalent cations such as Mg^2+^ or Mn^2+^. In native interactions, these ions form direct electrostatic bridges with the Asp residue of RGD-containing ligands, anchoring them firmly into the cleft. In our model, the 17-OH group of E_2_ projects toward this ion-stabilized region, suggesting that estrogen may exploit the MIDAS coordination environment to achieve stable binding, with the top-ranked docking pose based on the lowest binding free energy (ΔG ≈ −8.1 kcal/mol.)

Post-docking visualization was performed using PyMOL to generate 3D binding representations and LigPlot+ (v2.2) to generate 2D interaction diagrams. Interaction types were automatically annotated and manually verified, including hydrogen bonds, van der Waals, alkyl, and π-alkyl interactions. The integrin surface was colored according to hydrophobicity to illustrate the spatial orientation of estradiol within the cRGD-binding groove, indicating overlap with the canonical RGD-binding site near the MIDAS region.

The binding landscape reveals multiple stabilizing contacts, including van der Waals interactions, π-alkyl stacking, and hydrogen bonding with residues such as Asp1382, His752, Thr1432, Val1429, Cys813, Pro750, and Phe754. Hydrophobicity mapping shows that E_2_ embeds into a mixed chemical environment, engaging both hydrophilic (ion-rich) and hydrophobic surfaces of the cRGD pocket. This positioning mirrors the canonical RGD peptide but is achieved through steroidal contacts and the orientation of the hydroxyl groups.

This docking model provides mechanistic insight into how estrogen may directly engage the cRGD site of integrin αvβ3. By leveraging the ion-dependent MIDAS for binding, estradiol could potentially influence integrin-mediated signaling pathways and alter the migratory and invasive behavior of ER-positive breast cancer cells, extending the functional role of estrogen beyond classical ER-α signaling.

## 4. GPER-Dependent Effects of Estrogen

Notably, low nanomolar concentrations of bisphenol A (BPA) or E_2_ have been shown to diminish cisplatin-induced cytotoxicity in breast cancer cells, and these effects occur independently of classical ERs [49,50]. These findings suggest an extracellular binding site for E_2_ and BPA in both ER-α-positive and ER-α-negative breast cancer cells. Estrogen interacts with the GPER at the cell membrane, triggering rapid, non-genomic signaling pathways [51]. This interaction sequentially activates downstream effectors like adenylyl cyclase, cAMP, and MAPK to regulate cell proliferation and migration [52]. Additionally, estrogen activates a Gαi1/Gβ protein-dependent signaling pathway involving ER-α to the activation of c-Src and FAK. This results in the phosphorylation of paxillin, which regulates cell adhesion and motility, rapidly forming focal adhesion complexes at sites related to cell movement [53]. A selective ER modulator, raloxifene, inhibits estrogen-promoted cell adhesion and migration by targeting the FAK/paxillin/N-WASP signaling pathway [53]. GPER is also implicated in drug resistance. It reduces Bim proteins through the MAPK/ERK-TRIM2 signaling axis to promote tamoxifen-resistance in ER-α-positive breast cancer cells [54]. Furthermore, GPER is involved in the resistance to the CDK4/6 inhibitor palbociclib in ER-α-positive breast cancer [55]. Interestingly, E_2_ downregulates GPR30 mRNA expression via ER but not GPER to inhibit the proliferation of ER-α-positive breast cancer cells [56].

GPER is highly expressed in TNBC cell lines, such as MDA-MB-231 and SUM159, and has been implicated in mediating the proliferative effects of estrogens in ER-α-positive breast tumors [27]. High GPER expression is also observed in TNBC patients, linked to pro-metastatic pathways, and predictive of poor patient outcomes [57]. The estrogen-activated nongenomic signal transduction pathway, primarily dependent on ERK1/2 activation, plays a critical role in the viability and motility of TNBCs. This pathway is activated through GPER, which induces FAK activation by triggering Y397 FAK phosphorylation and increasing focal adhesion points in TNBCs [27]. Activating the estrogen/GPER/ERK signaling pathway through treatment with E_2_, the GPER-specific agonist G-1, or tamoxifen rapidly stimulated ERK1/2, but not Akt, in TNBC cell lines such as MDA-MB-468 and MDA-MB-436 cells. This pathway is associated with cell growth, survival, and apoptosis by upregulating the expression of cyclin A, cyclin D1, Bcl-2, and c-fos, which regulate the cell cycle, proliferation, and apoptosis. Moreover, the estrogen/GPER/ERK signaling pathway promotes migration and invasion in TNBCs [58]. The long non-coding RNA HOTAIR plays a crucial role in regulating cancer cell proliferation and invasion in breast cancer. Estrogenic GPER signaling increases the HOTAIR level by inhibiting miR-148a in ER-α-negative breast cancer [59]. However, HOTAIR is regulated by estrogen via ER-α in ER-α-positive breast cancer [59].

## 5. Integrin αvβ3-Dependent Effects of Estrogen

Integrin αvβ3 has been identified as a key mediator in thyroxine- and androgen-driven tumor progression in breast cancer cells. Although estrogen does not stimulate proliferation in ER-negative breast cancer cells, it has been shown to enhance pulmonary metastasis in ER-negative breast cancer [60]. Moreover, estrogen reverses the antiproliferative effects of integrin αvβ3 agonist heteronemin [61]. Furthermore, the activities of FAK and paxillin depend on integrin αv [62]. Treatment with CNTO 95, a fully human anti-integrin αv antibody, decreased the viability of breast tumor cells adhering to vitronectin in vitro. In addition to its inhibitory effect on breast tumor cell viability in vitro, CNTO 95 inhibited tumor cell adhesion, migration, and invasion. CNTO 95 treatment also induced the tyrosine dephosphorylation of FAK and the docking protein paxillin, which is crucial for recruiting structural and signaling molecules to focal adhesions [62,63]. These results suggest that CNTO 95 inhibits breast tumor cell growth, migration, and invasion by interrupting integrin αv-mediated focal adhesions and cell motility signals [62].

Furanodiene, a natural terpenoid isolated from Rhizoma Curcumae, exhibits anticancer effects by inhibiting angiogenesis and inducing reactive oxygen species production, DNA strand breaks, and apoptosis [64]. Furanodiene combined with doxorubicin blocked the invasion and migration of breast cancer MDA-MB-231 cells in vitro. The combined treatment inhibited the expression of integrin αv and β-catenin. It also inhibited the phosphorylation of paxillin, Src, FAK, p85, and Akt. Additionally, the combination treatment decreased matrix metalloproteinase-9 (MMP-9) expression [65]. However, furanodiene did not significantly alter the effects of doxorubicin on the tubulin cytoskeleton, as evidenced by no changes in the expression levels of RhoA, Cdc42, N-WASP, and α- and β-tubulin. Furthermore, competitive inhibition of integrin αvβ3 also suppresses the proliferation of triple-negative breast cancer cells. E_2_ downregulates the expression of integrin αvβ3, which is closely associated with the growth of breast cancer cells [66]. Notably, the overexpression of integrin αvβ3 in tumor-associated vasculature serves as a marker of poor prognosis in breast cancer [66].

The accumulation of integrin αvβ3 positively correlates with the overexpression of the growth factor heregulin, which is involved in breast cancer progression [67]. Estrogen induced a drastic downregulation of integrin αvβ3 expression (up to 50%) in ER-α-stably expressing breast cancer MDA-MB-231 cells (S30). After E_2_ treatment, S30 cells exhibit a specific decrease in cell growth and integrin αvβ3 levels, while the expression of other integrins remains unaffected. However, E_2_ does not alter the integrin expression in heregulin-overexpressing ER-α-negative MDA-MB-231 cells. The reduction in integrin αvβ3 levels induced by E_2_ is related to a decrease in the G2/M population of the cell cycle of S30 cells. Overexpression of αvβ3 is associated with loss of E_2_ dependence and antiestrogen response in highly invasive breast cancer cells [66]. These findings suggest a close relationship between E_2_ and integrin αvβ3 in breast cancer cells. However, no evidence shows estrogen via ER-β interacts with integrin αvβ3.

**Figure 2:**
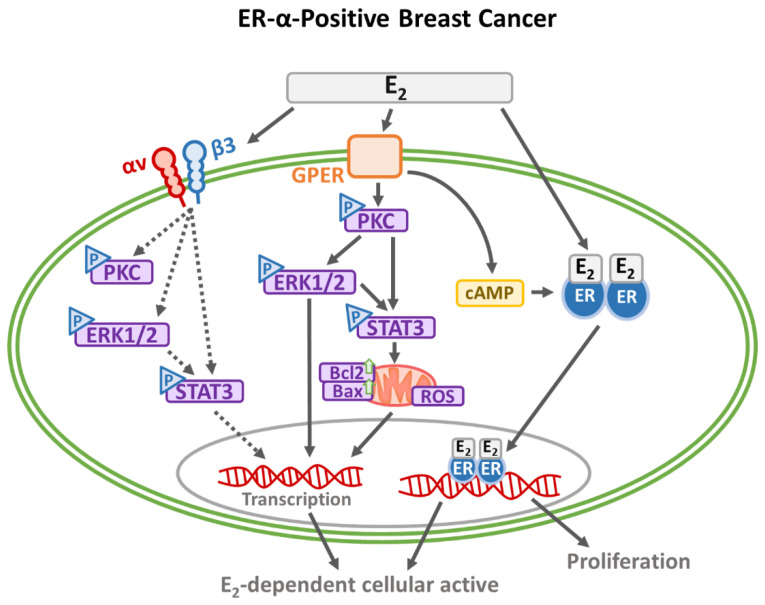
Signal transduction pathways induced by estrogen in ER-α-positive breast cancer cells. Estrogen activates both canonical and non-canonical signaling pathways in ER-positive breast cancer cells. In the canonical pathway, estrogen diffuses across the membrane, binds to ER-α monomers, induces dimerization, and translocates to the nucleus, where ER regulates gene expression by directly binding DNA or interacting with transcription factors. ERα activates downstream pathways involving MAPK, JAK/STAT, SRC, and PI3K. In the non-canonical pathway, extracellular estrogen binds to membrane-bound receptors, including GPER and integrin αvβ3, initiating STAT3 and Ras-ERK1/2 signaling cascades. These pathways contribute to cell proliferation, migration, and metastasis. Notably, integrin αvβ3-mediated signaling enhances ERK1/2 and STAT3 activation and may upregulate integrin αvβ3 expression itself, suggesting a feedback loop that promotes tumor progression. Solid black arrows represent activated signal, dashed black arrows indicate possible stimulatory signals, and blank arrows denote increased expression.

**Figure 3:**
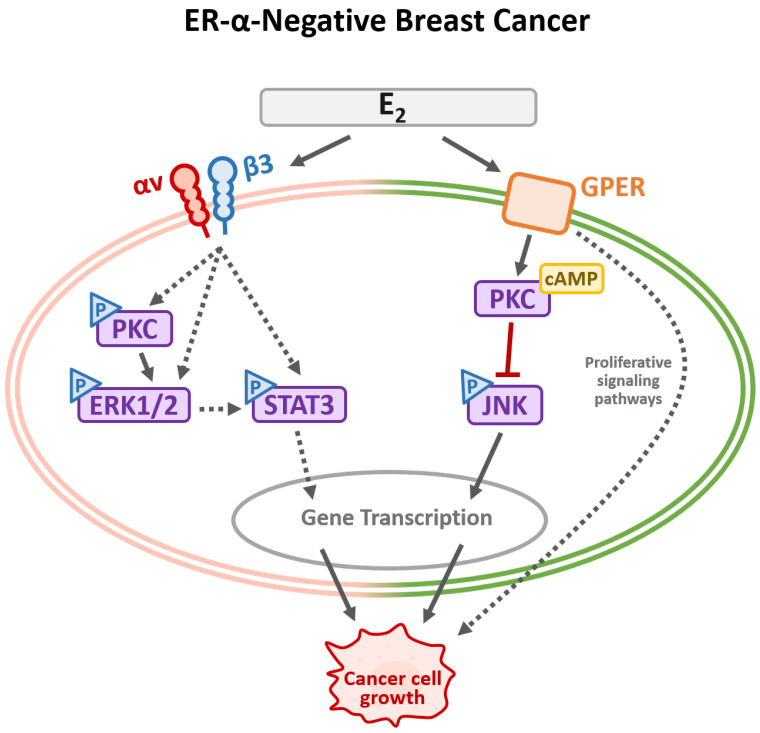
Signal transduction pathways induced by estrogen in ER-α-negative breast cancer cells. The extracellular estrogens bind to the plasma membrane GPER or integrin αvβ3 receptor on the cell surface in ER-α-negative breast cancer cells, promoting signal transduction pathways. E_2_ via GPER recruits cAMP to bind with JNK and inhibit JNK activation. On the other hand, a signal via activated integrin αvβ3 stimulates the activation of ERK1/2 and STAT3. Those signals are vital in gene expression in cancer cell growth, promoting migration or other activities. Solid black arrows represent activated signal and dashed black arrows indicate possible stimulatory signals.

An integrated overview of estradiol-triggered signal transduction mechanisms in ER-α-positive and ER-α-negative breast cancer cells was illustrated in Figure 2 and Figure 3. E_2_ contributes significantly to breast cancer development by engaging both nuclear and membrane-associated receptors, thereby initiating a spectrum of intracellular signaling events.

In ER-α-positive cells, E_2_ traverses the lipid bilayer and binds to ER-α monomers, which dimerize and relocate to the nucleus. These receptor complexes modulate transcriptional activity either through direct interaction with estrogen-responsive DNA elements or indirectly via co-regulatory transcription factors. This classical ER-α signaling axis activates key oncogenic pathways, including MAPK, JAK/STAT, SRC, and PI3K, which collectively enhance tumor cell proliferation, survival, and metastatic behavior. In addition to this genomic route, E_2_ activates non-genomic signaling through membrane-localized receptors such as GPER and integrin αvβ3. These receptors initiate rapid signaling cascades, specifically PI3K/Akt, STAT3, and Ras-ERK1/2, which regulate gene expression and promote cellular motility and invasion. Integrin αvβ3 activation stimulates ERK1/2 and STAT3, facilitating cytoskeletal remodeling and potentially upregulating its expression in response to E_2_, thereby reinforcing a pro-tumorigenic feedback loop [68].

In ER-α-negative breast cancer cells lacking functional nuclear ER-α, E_2_ still exerts a biological influence through membrane-bound receptors. GPER activation leads to the recruitment of cyclic AMP (cAMP), which binds to JNK and suppresses its phosphorylation, thereby modulating apoptotic and stress-related responses. Simultaneously, integrin αvβ3 engagement activates ERK1/2 and STAT3 signaling, contributing to transcriptional changes that support tumor cell growth, migration, and invasion. These non-genomic pathways are particularly relevant in triple-negative breast cancer subtypes, where classical ER signaling is absent, but estrogen responsiveness persists via alternative receptor systems. Together, these findings underscore the complexity of E_2_-mediated signaling in breast cancer and highlight the dual roles of genomic and non-genomic pathways in driving disease progression across molecular subtypes.

On the other hand, the interaction between GPER and integrin is complicated. GPER does not always directly connect to integrin, as there is a lack of evidence to show that integrin αvβ3 directly interacts with GPER. However, Zhiguo Sheng et al. discovered that membrane G protein-coupled estrogen receptor 1 (GPER) and integrin αvβ3, along with their respective signal pathways, participate in the induction of male germ cell proliferation and thyroid transcription disruption by low-dose BPA [69]. Francesca Cirillo et al. described that connective tissue growth factor CTGF is overexpressed in TNBCs and mediates the migration and invasion through the integrin αvβ3/ERK pathway [70] as well as aerobic glycolysis via the FAK/Src/NF-κB/Glut3 axis [24]. Microarray transcriptome analysis by Francesca Cirillo et al. indicated that CTGF is a main target gene of GPER [71]. GPER knocked-down MDA-MB-231 cells display a reduced expression and secretion of CTGF [72]. Studies also indicate that activated GPER can lead to integrin α5β1 activation through Gβγ subunits [73]. Alternatively, integrin α5β1 regulates integrin αvβ3-mediated extracellular signal-regulated kinase activation [74]. GPER/EGFR/ERK signaling promotes β1-integrin expression and sequentially activates downstream kinases, contributing to cancer-associated fibroblast-induced cell migration and epithelial–mesenchymal transition in MCF-7R cells [75]. GPER may contribute to tamoxifen resistance in a β1-integrin-dependent manner, interacting with the tumor microenvironment [75].

**Figure 4:**
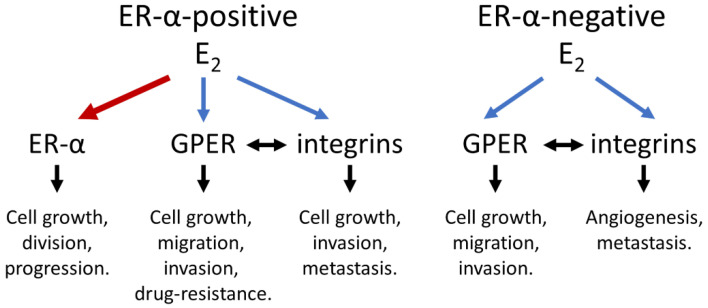
Schematic of Estrogen interactions with ER-α, GPER, and integrin in breast cancer cells. In ER-positive breast cancer, estradiol (E_2_) promotes cancer growth and progression through genomic action mediated by binding to estrogen receptor alpha (ER-α). E_2_ also activates non-genomic signaling pathways via G protein-coupled estrogen receptor (GPER) and integrins, contributing to cancer cell proliferation, metastasis, and invasion. In ER-negative breast cancer, E_2_ enhances cancer growth, metastasis, and invasion predominantly through non-genomic mechanisms involving GPER and integrins. Red arrows indicate genomic actions, blue arrows denote non-genomic actions, and the black arrows represent active mechanisms.

The biological activities of estrogen’s interaction with ER-α, GPER, and integrin are summarized in Table 2. Furthermore, the interactions among estrogen, GPER, and integrin are schematized in Figure 4.

Integrin αvβ3 plays a crucial role in breast cell growth, adhesion, and migration [83]. It appears that E_2_ interacts with integrin αvβ3 by binding to the RGD-binding site. Estrogen concentration regulates the expression of integrin αvβ3 in breast cancer cells [66]. Estrogen also modulates the degradation of p53 via ubiquitin-specific peptidase 10 (USP10) in breast cancer cells [84]. USP10 post-translationally regulates protein expressions of integrins, including αv, β1, and β5, thereby influencing the surface expression of integrin αvβ3 [85]. USP10 stimulates proliferation in ER-α-positive breast cancer cells [84] and ER-α-negative breast cancer cells by enhancing the stability of TCF4 protein [86]. USP10 has been shown to positively correlate with the Yes1-associated transcriptional regulator (YAP1 or YAP), thereby promoting breast cancer progression and metastasis [87]. YAP1 is highly expressed and hypomethylated in human breast cancer tissues. In ER-α-positive breast cancer, YAP/TAZ is required for estrogen-induced transcription and breast cancer growth [88]. Interestingly, YAP may also act as a tumor suppressor in ER-α-positive breast cancer. Increased YAP1 expression is negatively associated with DNA methyltransferase 3B (DNMT3B) expression [89]. E_2_ via ER-α modulates YAP1 expression by inducing hypomethylation of its promoter region through the downregulation of DNMT3B in ER-α-positive breast cancer cells [89]. However, Hippo pathway transcription factor TEAD physically interacts with ER-α to increase its promoter/enhancer occupancy [90]. In contrast, YAP inhibits ERα/TEAD interaction, decreases ER-α occupancy on its target promoters/enhancers, and promotes ER-α degradation by the proteasome [90]. Additionally, YAP inhibits hormone-independent transcription of the ER-α gene [91]. Conversely, YAP promotes the growth of ER-α-negative breast cancer. YAP activation is positively regulated by integrin αvβ3 [92], as activated integrin αvβ3 triggers ERK-mediated YAP phosphorylation, translocating YAP to the nucleus [93]. This translocation stimulates the expression of ankyrin repeat domain 1 (ANKRD1), connective tissue growth factor (CTGF), and inhibin βA (INHBA). Additionally, YAP induces TEAD-dependent transcription of thrombospondin 1 (THBS1), which drives focal adhesion and invasion. SUMOylation of OTUB2 by EGF and KRAS stabilizes YAP in the cytosol through deubiquitination, further regulating YAP activation. Alternatively, studies indicated that Hippo/MST1/2 inhibition or YAP activation can suppress the ER-α transcriptional program and ER-α-positive breast cancer growth [90]. Moreover, E_2_ treatment also stimulates YAP1 expression in ER-α-negative breast cancer cells, such as MDA-MB231 and SKBR3 [94]. The roles of YAP in breast cancer cells are summarized in Table 3.

We have summarized the signaling sections to delineate three distinct and coherent pathways:

(1) ERα-positive, genomic signaling

In ERα-positive breast cancer, estrogen binds to nuclear estrogen receptor alpha (ERα), initiating transcriptional regulation of target genes involved in proliferation and survival. This classical genomic pathway is well-characterized and central to hormone-responsive tumor biology.

(2) ERα-positive/negative, non-genomic signaling via GPER

Estrogen can also activate rapid, non-genomic signaling through the G protein-coupled estrogen receptor (GPER), which is expressed in both ERα-positive and ERα-negative breast cancers. GPER-mediated signaling involves second messengers and kinase cascades, contributing to cell proliferation, migration, and resistance mechanisms.

(3) Integrin αvβ3–linked, non-genomic signaling (FAK/ERK/PI3K, STAT3, YAP)

Integrin αvβ3 serves as a membrane-associated signaling hub that can be activated by estrogen in a non-genomic manner. This pathway engages focal adhesion kinase (FAK), ERK, PI3K/AKT, and STAT3, leading to downstream effects on cell motility and survival. Notably, YAP (Yes-associated protein) is a key downstream effector in this axis. Under normal conditions, YAP is phosphorylated and retained in the cytoplasm or degraded. In breast cancer, however, YAP translocates to the nucleus and functions as a transcriptional co-activator, promoting genes associated with tumor progression. Integrin αvβ3 activates YAP via FAK signaling, and FAK itself is a critical upstream regulator of YAP activity. Additionally, increased matrix stiffness—a hallmark of the breast tumor microenvironment—enhances FAK activation, which facilitates YAP nuclear translocation and activity, thereby promoting cell migration and invasion. The PI3K/AKT pathway also positively regulates YAP through multiple mechanisms in mammary tumorigenesis.

## 6. Conclusions

In conclusion, estrogen orchestrates both genomic and non-genomic signaling pathways that collectively regulate the behavior of breast cancer cells. Integrin αvβ3, traditionally recognized for its role in signal transduction and extracellular matrix interactions, has been identified as a membrane-associated estrogen receptor in both ER-α-positive and ER-α-negative breast cancer cells. While ER signaling remains central to endocrine therapy in ER-α-positive breast cancer, integrin αvβ3-mediated non-genomic estrogen signaling—particularly via GPER—contributes to tumor survival and chemoresistance in ER-α-negative and TNBC subtypes. As such, integrin αvβ3 represents a promising biomarker and therapeutic target, particularly in aggressive tumors that are unresponsive to conventional hormone therapies. Elucidating the molecular crosstalk between estrogen- and integrin-mediated pathways may pave the way for more precise, subtype-specific interventions in breast cancer. The interplay between E_2_, ER-α, and integrin αvβ3 has significant therapeutic implications. Although integrin αvβ3 has been a research focus for many years, and some preclinical studies have shown encouraging results in targeting this integrin, anti-integrin therapies have repeatedly failed in clinical trials [102,103]. Consequently, other integrins are now being explored as potentially better targets [104]. Molecular docking studies have identified the Asp residue of the cRGD ligand as a critical binding domain that mediates the estrogen–integrin αvβ3 interaction, thereby initiating downstream non-genomic signaling cascades. These pathways converge on nuclear transcriptional regulators such as YAP, which plays different roles in ER-αand ER-α-negative breast cancer cells. Notably, ERK phosphorylation facilitates the nuclear translocation of YAP and the transcriptional activation of oncogenic targets. Additionally, YAP enhances the expression of PD-L1, thereby mitigating tumor progression and immune evasion in triple-negative breast cancer [94]. YAP regulates gene expression involved in proliferation, invasion, metastasis, and drug resistance, and its activity is tightly regulated by integrin αvβ3. Taken together, these findings suggest that integrins, particularly those influencing YAP signaling, may serve as viable therapeutic targets in breast cancer.

## Figures and Tables

**Table 2:** Estrogen interacts with ER-α, GPER, and integrin in breast cancer cells.

	ER-α-Positive Cells	ER-α-Negative Cells
Receptor		
Primary ERs	Nuclear ER-α and ER-β; minor role for membrane ERs.	
Membrane GPER	Membrane GPER	Membrane GPER
Role of E_2_	Genomic (regulating gene expression) and non-genomic (rapid signaling from membrane ERs).	Non-genomic, mediated by GPER activation.
Functions of Membrane GPER	To promote tumor progression by stimulating cell growth, migration, and invasion [51]. To link to tamoxifen resistance [54] or palbociclib resistance [55]. To activate apoptosis [76]	To stimulate cancer growth, migration, and invasion via highly expressed GPER non-genomic signaling [27,58]. It is associated with pro-metastatic pathways and predictive of poor patient outcomes [57].
Integrin specificity	E_2_ proposed to bind to integrin αvβ3 directly based on molecular docking modeling, although its binding to integrin αvβ3 has been experimentally demonstrated in other cell types. [12]. Membrane-bound ER-α crosstalks with kinases and integrin signaling complexes [77].	E_2_ proposed to bind to integrin αvβ3 directly based on molecular docking modeling, although its binding to integrin αvβ3 has been experimentally demonstrated in other cell types [12]. GPER activation leads to integrin activation (e.g., α5β1) through Gβγ subunits. The subsequent RTK-integrin crosstalk influences a variety of adhesion molecules [78].
Interaction with integrins	E_2_/ER-α signaling regulates integrin expression genomically [79] or activates non-genomic activities through crosstalk with pathways such as SRC/MAPK [80].	E_2_/GPER activation transactivates RTKs like EGFR, which signal through integrins and associated complexes [81,82]. GPER can also directly activate integrins [75]. E_2_ directly binds with integrin αvβ3 to activate signals.
Signaling transduction	The context-dependent integration of multiple ER-α pathways modulates integrin expression, clustering, and cytoskeletal interactions. ER-α promotes proliferation.	GPER establishes an RTK-integrin signaling axis that drives proliferation and survival in a manner distinct from the classical nuclear ER pathway.

The interactions among estrogen, GPER, and integrin are schematized in Figure 4.

**Table 3:** Effect of YAP in ER-α-positive and ER-α-negative breast cancer cells.

Aspect	ER-α-Positive Breast Cancer	ER-α-Negative Breast Cancer
Primary Function	YAP/TAZ is required for estrogen-induced transcription for breast cancer growth [88].	Reduced metastasis suppressor, KIBRA, promotes the oncogenic function of YAP/TAZ in growth [95].
	YAP/TAZ is required for the transcriptional repression of ESR1 (ER-α) [91].	Tumor suppressor SYNPO2 inhibits YAP/TAZ activity to suppress metastasis in triple-negative breast cancer [96].
	YAP disrupts a TEAD-ER-α signaling axis to inhibit ERα and ER-α-positive breast cancer growth [90].	YAP/TAZ/TEAD associated with AP-1 at enhancers drives oncogenic growth of breast cancer [97].
	Overexpression of YAP, CTGF, and CYR61 induces transcriptional repression of ER-α and tamoxifen resistance in breast cancer [98].	Interaction of ZEB1 and YAP/TEAD stimulates the breast cancer cell aggressiveness [99].
Clinical Association	High YAP is associated with a better prognosis and a more favorable response to tamoxifen.	High YAP correlates with poor prognosis, increased metastasis, and therapy resistance.
Regulation by Estrogen	Estrogen and ER-α upregulate YAP1 expression by decreasing DNMT3B and causing promoter hypomethylation [89].	E_2_ treatment stimulates YAP1 expression in MDA-MB231 and SKBR3 ER-α-negative breast cancer cells [94]. Other pathways, such as cell density changes, loss of cell contact, or mutations, often drive YAP activation. [100,101].

## Data Availability

No new data were created or analyzed in this study.

## Abbreviations

BPA	Bisphenol A
CCN	Cellular communication network
Cdc42	Cell division control protein 42
cRGD	Cyclic RGD
CTGF	Connective tissue growth factor
E_2_	Estradiol (17β-Estradiol)
ECM	Extracellular matrix
ER	Estrogen receptor
ERK1/2	Extracellular signal-regulated kinase 1/2
FAK	Focal adhesion kinase
Glut3	Glucose transporter 3
GPER	G protein-coupled estrogen receptor
IL	Interleukin1
JAK	Janus kinase
MAPK	Mitogen-activated protein kinase
NF-κB	Nuclear factor kappa-light-chain-enhancer of activated B cells
N-WASP	Neuronal Wiskott–Aldrich syndrome protein
PI3K	Phosphatidylinositol 3-kinase
Rac1	Ras-related C3 botulinum toxin substrate 1
Ras	Rat sarcoma
RhoA	Ras homolog family member A
RGD	Arg-Gly-Asp
RNF31	RING finger protein 31
Shc	Src homology and collagen
TEAD	TEA domain transcription factor
TGF-β	Transforming growth factor-β
TH	Thyroid hormone
TAK1	Activated kinase 1
VEGF	Vascular endothelial growth factor
UTMD	Ultrasonic targeted microbubble destruction
YAP	Yes-associated protein YB-1: Y-box binding protein 1
